# Surveillance of Rodent Pests for SARS-CoV-2 and Other Coronaviruses, Hong Kong

**DOI:** 10.3201/eid2802.211586

**Published:** 2022-02

**Authors:** Elliott F. Miot, Brian M. Worthington, Kar Hon Ng, Lucy de Guilhem de Lataillade, Mac P. Pierce, Yunshi Liao, Ronald Ko, Marcus H. Shum, William Y. Cheung, Edward C. Holmes, Kathy S. Leung, Huachen Zhu, Leo L. Poon, Malik J. Peiris, Yi Guan, Gabriel M. Leung, Joseph T. Wu, Tommy T. Lam

**Affiliations:** University of Hong Kong State Key Laboratory of Emerging Infectious Diseases, Hong Kong, China (E.F. Miot, B.M. Worthington, K.H. Ng, L. de Guilhem de Lataillade, M.P. Pierce, Y. Liao, M.H. Shum, W.Y. Cheung, H. Zhu, Y. Guan, T.T. Lam);; University of Hong Kong School of Public Health, Hong Kong (E.F. Miot, B.M. Worthington, K.H. Ng, L. de Guilhem de Lataillade, M.P. Pierce, Y. Liao, R. Ko, M.H. Shum, W.Y. Cheung, H. Zhu, L.L. Poon, M.J. Peiris, Y. Guan, G.M. Leung, J.T. Wu, T.T. Lam);; University of Hong Kong HKU-Pasteur Research Pole, Hong Kong (E.F. Miot, L. de Guilhem de Lataillade, L.L. Poon, M.J. Peiris);; Centre for Immunology & Infection Limited, Hong Kong (E.F. Miot, L. de Guilhem de Lataillade, L.L. Poon, M.J. Peiris, T.T. Lam);; Shantou University/University of Hong Kong Guangdong-Hongkong Joint Laboratory of Emerging Infectious Diseases, Shantou, Guangdong, China. (B.M. Worthington, W.Y. Cheung, H. Zhu, Y. Guan, T.T. Lam);; EKIH (Gewuzhikang) Pathogen Research Institute, Shenzhen City, Guangdong, China. (B.M. Worthington, W.Y. Cheung, H. Zhu, Y. Guan, T.T. Lam);; Laboratory of Data Discovery for Health Limited, Hong Kong, China (W.Y. Cheung, E.C. Holmes, K.S. Leung, H. Zhu, Y. Guan, G.M. Leung, J.T. Wu, T.T. Lam);; Marie Bashir Institute for Infectious Diseases and Biosecurity, University of Sydney School of Biological Sciences, and Sydney Medical School, Sydney, New South Wales, Australia (E.C. Holmes)

**Keywords:** COVID-19, SARS-CoV-2, severe acute respiratory syndrome coronavirus 2, coronavirus disease, rodents, serosurveillance, urban public health, viruses, zoonoses, respiratory infections, Hong Kong, China

## Abstract

We report surveillance conducted in 217 pestiferous rodents in Hong Kong for severe acute respiratory syndrome coronavirus 2 (SARS-CoV-2). We did not detect SARS-CoV-2 RNA but identified 1 seropositive rodent, suggesting exposure to a virus antigenically similar to SARS-CoV-2. Potential exposure of urban rodents to SARS-CoV-2 cannot be ruled out.

Severe acute respiratory syndrome coronavirus 2 (SARS-CoV-2) was first identified in Wuhan, China, in late 2019 (*1*) and soon spread globally. Although its zoonotic origin remains unclear, animal species potentially susceptible to reverse-zoonotic transmission from humans have been identified (e.g., cats, dogs, minks, deer), some of which (e.g., mink) might maintain the virus and pose a risk of future spillback to humans (*2*,*3*). Domestic animals and urban wildlife are of particular concern (*4*) because of their potential exposure to viruses shed within urban environments. Analysis of the angiotensin-converting enzyme 2 (ACE2) receptor across diverse vertebrates suggests a potentially wide breadth of SARS-CoV-2–susceptible mammal host species (*5*).

The rapid transmission and adaptation of SARS-CoV-2 in humans has been characterized by the evolution of variants of concern (VOCs). Several VOCs, particularly the Alpha (B.1.1.7), Beta (B.1.351), and Gamma (P.1) variants, have convergently evolved an amino acid residue change in the receptor binding domain of the spike protein (N501Y) that was also observed following serial passage of SARS-CoV-2 in BALB/c mice (*6*). Recent in vitro and in vivo experiments have demonstrated that these VOCs are capable of infecting laboratory rats and mice (*7*; Montagutelli X et al., unpub. data, https://doi.org/10.1101/2021.03.18.436013). Such evolutionary processes indicate a possible risk for reverse-zoonotic transmission of VOCs into urban rodents.

We hypothesized that locations with positive SARS-CoV-2 detection in sewage could also serve as key surveillance targets for potential exposure of pestiferous urban rodents to SARS-CoV-2 shed into the environment. We conducted sewage surveillance in Hong Kong to identify hidden infections and localized outbreaks of SARS-CoV-2 (*8*) during the fourth wave of COVID-19 in Hong Kong (Appendix).

During February 3–May 12, 2021, we sampled 217 rodents (*Rattus* spp.), 193 live-trapped rodents and 24 found dead near collection sites (Appendix Table 1). We collected 189 *R. norvegicus* and 28 *R. tanezumi* rats from 8 districts, the majority (n = 186) from Sham Shui Po, Yau Tsim Mong, and Kowloon City (Figure), where SARS-CoV-2 positive sewage has been reported.

**Figure:**
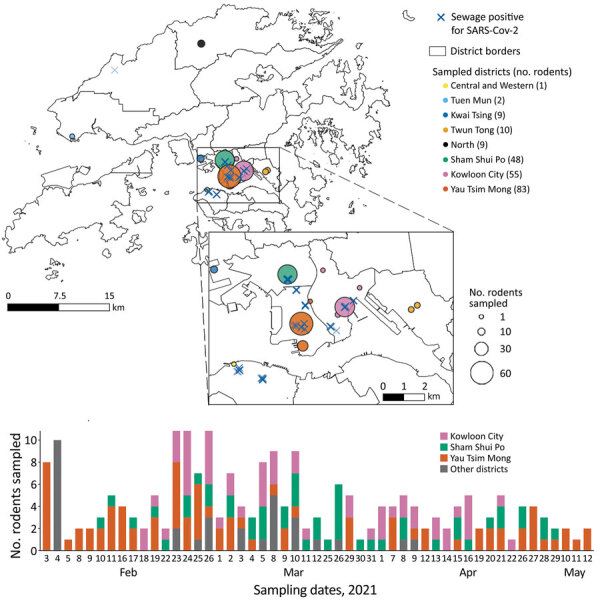
Surveillance of rodents for SARS-COV-2 conducted February–May 2021 in Hong Kong. A) Sampling sites, with number of rodents sampled and sewage testing positive for SARS-COV-2. Each circle represents a sampling location, color-coded by district and sized proportional to the number of captured rodents. Blue crosses represent locations where sewage was reported positive for SARS-COV-2during January 19–March 30, 2021. B) Number of sampled rodents, by collection dates and district. SARS-COV-2, severe acute respiratory syndrome coronavirus 2

We found samples from 1,702 swabs and tissues from 217 rats negative for SARS-CoV-2 by real-time quantitative PCR and 15 from 9 rats positive for murine alphacoronaviruses and betacoronaviruses using PCR and phylogenetic analysis (Appendix Table 2, Figure 1). Using ELISA, we identified 1 of 213 rodent serum samples from an *R. norvegicus* rat collected in Yau Ma Tei seropositive for SARS-CoV-2 (Table; Appendix Figure 2) and 11 samples inconclusive; only 1 of 2 replicates from 8 samples gave a positive absorbance result, and 1 or both replicates from 3 samples gave a borderline absorbance (Table; Appendix Figure 2). The unambiguously positive sample, from rat no. 213, was confirmed positive in surrogate virus neutralization testing (sVNT; 31.7% inhibition), but negative by plaque-reduction neutralization test (PRNT_90_; <10 titers for 90% reduction). All 11 inconclusive samples were negative (<20% inhibition) by sVNT. As a pre–COVID-19 biological control to test for cross-sensitivity, 50 rodent serum samples collected in 2008 were examined by ELISA; none exhibited an unambiguously positive result.

**Table:** Information on rodents with unambiguous (n = 1) or inconclusive (n = 11) positive serum samples in ELISA testing in study of surveillance of rodent pests for severe acute respiratory syndrome coronavirus 2 and other coronaviruses, Hong Kong*

Animal code	*Rattus* species	Collection date	District	ELISA A/CO		sVNT, inhibition, %
				1st replicate	2nd replicate	
Rat-027	*R. tanezumi*	Feb 11	Sham Shui Po	0.019	0.855	1.281
Rat-069	*R. norvegicus*	Feb 24	Kowloon City	0.837	0.964	0.991
Rat-070	*R. norvegicus*	Feb 24	Kowloon City	1.199	0.472	–2.128
Rat-073	*R. tanezumi*	Feb 25	Yau Tsim Mong	1.445	0.033	2.224
Rat-076	*R. norvegicus*	Feb 25	Sham Shui Po	1.644	0.027	1.136
Rat-089	*R. norvegicus*	Mar 1	Yau Tsim Mong	1.324	–0.041	1.209
Rat-090	*R. norvegicus*	Mar 1	Yau Tsim Mong	1.636	–0.027	–0.532
Rat-096	*R. norvegicus*	Mar 2	Yau Tsim Mong	0.934	–0.007	3.748
Rat-097	*R. norvegicus*	Mar 2	Yau Tsim Mong	1.592	0.013	–4.666
Rat-098	*R. tanezumi*	Mar 2	Sham Shui Po	1.920	–0.724	–2.466
Rat-102	*R. norvegicus*	Mar 3	Kwai Tsing	0.992	–0.499	0.145
Rat-213†	*R. norvegicus*	May 10	Yau Tsim Mong	13.643	14.497	31.7

*A/CO was interpreted as negative if <0.9, borderline if 0.9–1.1, and seropositive if >1.1, according to manufacturer instructions. Serum was considered unambiguously positive if both replicates were seropositive. Positive cutoff for sVNT was 20% inhibition, as described elsewhere (*9*). A/CO, absorbance cutoff; sVNT, surrogate virus neutralization test. †Positive in both ELISA and sVNT.

Our rodent surveillance in Hong Kong revealed potential exposure to SARS-CoV-2, and although viral RNA was not detected, this could be a limitation of sample size if prevalence of active infection was low. One serum sample showed positive ELISA and sVNT results but negative PRNT_90_ results. Previous research demonstrated that the sVNT used in our study has >98.8% specificity and sensitivity without cross-reaction to alphacoronaviruses and murine betacoronavirus (*9*). Some sVNT-positive COVID-19–confirmed patients did not meet the threshold for positivity by PRNT_90_ (*9*). This finding suggests that the seropositive result for SARS-CoV-2 or a closely related virus in the brown rat was unlikely to be attributable to past exposure to murine alphacoronaviruses or betacoronaviruses. 

During our study period, SARS-CoV-2 infection was reported in several imported and local human cases in multiple locations and in multiple sewage results. Before December 2020, SARS-CoV-2 locally circulating in Hong Kong predominantly carried 501N with presumably lower rodent infectivity; however, during our study period, Hong Kong reported many imported cases of SARS-CoV-2 variants, including B.1.1.7 and B.1.351, carrying 501Y, which has been demonstrated in mouse experiments to be a critical genetic adaptation (*6*). These imported cases might disseminate virus into the environment near quarantine hotels, presenting an increased risk of spillover into urban rodent populations and requiring enhanced biosecurity to limit potential exposure to urban rodents or other susceptible animals. Our finding of potential SARS-CoV-2 exposure in a pestiferous rat highlights the need for sustained monitoring of rodent populations to rapidly detect spillover events and subsequently put in place timely interventions (e.g., disinfestation using trapping and pesticide) to prevent potential establishment of new reservoirs.

Appendix Additional information on surveillance of rodents for severe acute respiratory syndrome coronavirus 2 in Hong Kong.
