# Supplementary material for: Surveillance of Rodent Pests for SARS-CoV-2 and Other Coronaviruses, Hong Kong
Source: Emerg Infect Dis. 2022 Feb;28(2):467–70. doi: 10.3201/eid2802.211586 (PMC8798707; doi:10.3201/eid2802.211586)
Supplement: Appendix — Additional information on surveillance of rodents for severe acute respiratory syndrome coronavirus 2 in Hong Kong. [file 21-1586-Techapp-s1.pdf]

Article DOI: https://doi.org/10.3201/eid2801.211586

# Surveillance of Rodent Pests for SARS-CoV-2 and Other Coronaviruses in Hong Kong

## Appendix

## Materials and Methods

### Sample Collection

Rodents from the genus *Rattus* (determined by morphology and confirmed using DNA barcoding) were trapped and sampled as part of rodent surveillance conducted during February–May 2021, in collaboration with the Food and Environmental Hygiene Department (FEHD) of Hong Kong SAR. Additional traps were placed in back alleys close to known SARS-CoV-2–positive sewage sites in Sham Shui Po, Yau Tsim Mong, and Kowloon City districts (Figure). Live-trapped rodents (n = 193) were euthanized with an overdose of isoflurane. Samples for pathogen surveillance were collected post-mortem. Rodents found dead near the sampling sites were also collected (n = 24).

Blood was collected by cardiac puncture and swab samples including body surface, oropharyngeal, and rectal swabs were collected in duplicate for all subjects. For rodents captured alive, a full necropsy was performed to collect most of the major organs (i.e., lymph nodes, heart, lung, trachea, liver, spleen, small and large intestine, kidney, bladder, and brain). When available, urine, feces, ectoparasites, and endoparasites were also collected. For dead rodents, except for 4 specimens, blood was collected in the thoracic cavity as well as the whole heart after a partial necropsy.

Blood samples were collected in CAT serum clot activator coated tubes to retrieve serum. In the case of dead rats, when possible, 1mL of phosphate buffer saline 1X was added to the blood and heart in CAT serum activator coated tube. Swab samples were collected in virus transport media (VTM) containing M199 media, antimicrobials, antifungal, bovine serum albumin and stabilizers previously described (*1*). Tissue samples were collected using standard

sterile techniques in microbiological practice and stored in VTM as well as in RLT lysis buffer (QIAGEN). Samples were transported on ice for same-day processing or dry ice to the State Key Laboratory of Emerging Infectious Diseases at the University of Hong Kong where further sample processing was conducted. The research protocol was approved by the University of Hong Kong Committee on the Use of Live Animals in Teaching and Research (CULATR 5657–21).

### RNA Extraction

RNA was extracted from swab (n = 651), urine (n = 94), and blood samples (n = 194, the first 23 were not extracted) using QIAamp Viral RNA Mini Kit (QIAGEN, https://www.qiagen.com), and from tissue samples (i.e., lymph nodes, lung, trachea, small intestines, n = 558) using RNeasy Plus Micro Kit (QIAGEN). Both kits were used according to manufacturer instructions except for the elution step. Elution was performed twice with 30 μL buffer AVE for QIAamp or RNase-free water for RNeasy with 5 min incubation at room temperature each time.

### SARS-CoV-2 Detection

The COVID-19 Real-Time PCR Kit (Chaozhou Hybribio Biochemistry Ltd, http://hybribio.com) was used for quantitative reverse transcription PCR with multiple fluorescence detection channels including FAM targeting SARS-CoV-2 ORF1ab, HEX targeting SARS-CoV-2 N region, and Cy5 targeting B2M gene as an internal control. We confirmed that this method was able to detect representative human SARS-CoV-2 circulating in Hong Kong during our sampling (WHP-4212) as well as from 1 imported case harboring the N501Y mutation (WHP-4238), which yielded a cycle threshold value <30.

### Universal Coronavirus Detection

The presence of other coronaviruses was assessed by a 2-step RT-PCR reaction to generate a 442 bp amplicon using universal coronavirus primers (UniCoV) targeting the most conserved region of the RNA-dependent RNA polymerase gene (CorUniF: 5′-ATGGGTTGGGATTATCCTAAGTGTGA-3′, CorUniR2: 5′-CATCATCAGATAGAATCATCATAG-3′, and CorUniR3: 5′-CCATCATCAGATAGAATCATCAT-3′ (*1*). Total RNA was reverse transcribed into complementary DNA (cDNA) using PrimeScript RT Master Mix (TaKaRa,

https://www.takarabio.com) using the following program: 15 min at 37°C followed by 5 sec at 85°C. The 20-µL reaction volume contained 4 µL of 5X PrimeScript RT Master Mix (Perfect Real Time) and 16 µL of RNA extract. The cDNA was subsequently amplified using AmpliTaq Gold DNA polymerase (ThermoFisher Scientific, https://www.thermofisher.com). The 25-µL reaction volume contained 2.5 µL 10x PCR Gold buffer, 1.5 µL $MgCl_2$ (25 mmol), 0.5 µL dNTP mix (10 mmol), 0.5 µL of forward primer CorUniF, 0.25 µL of reverse primers CorUniR2 and CorUniR3, 0.25 µL AmpliTaq Gold DNA polymerase (5 U/µL), and 1 µL of cDNA template. Amplification was performed using the following program: 5 min at 95°C, 45 cycles of 30 sec at 95°C, 30 sec at 48°C, and 45 sec at 72°C, followed by a final extension step of 5 min at 72°C. Amplicons were visualized by electrophoresis on a 1.5% agarose gel. PCR positive samples were purified using Expin PCR SV kit (GeneAll, https://www.pcr-lab-products.com) following manufacturer instructions and sequenced using an ABI 3730xl DNA Analyzer at the Centre for PanorOmic Sciences (http://www.med.hku.hk/en/research/facilities-and-services/cpos) (CPOS) to confirm the presence and identity of coronaviruses using BLASTn search (https://blast.ncbi.nlm.nih.gov/Blast.cgi) against the nucleotide database in GenBank. The amplicon sequences were submitted to GenBank under accession numbers OK018140 – OK018153.

**DNA Extraction and Rodent Species Identification**

DNA was extracted from the ear tissue of each animal using the DNeasy Blood & Tissue Kit (QIAGEN). Conventional PCR was used to amplify a 708 bp region of the mitochondrial cytochrome c oxidase subunit I (COI) gene (Rat-COI-F: 5′-CGTTGACTMTTTTCAACYAACCAC-3′, Rat-COI-R 5′-CRTGTGARATAATTCCAAAYCCTGG-3′) to confirm the species of each animal. If DNA extracted from ear tissue failed to yield an amplicon, cDNA from swabs or tissue samples were used as input for PCR. Barcoding of the COI region was performed using AmpliTaq Gold DNA polymerase (ThermoFisher Scientific) in a reaction volume of 25 µL containing 2.5 µL 10x PCR Gold buffer, 1.5 µL $MgCl_2$ (25 mmol), 0.5 µL dNTP mix (10 mmol), 0.5 µL of forward primer Rat-COI-F, 0.5 µL of reverse primer Rat-COI-R, 0.25 µL AmpliTaq Gold DNA polymerase (5 U/µL), and 2 µL of DNA or cDNA template. The barcoding PCR was performed using the following thermocycling program: 5 min at 94°C, 40 cycles of 30 sec at 94°C, 40 sec at 54°C, and 60 sec at 72°C followed by a final extension step of 10 min at 72°C. Amplicons were

visualized by electrophoresis on a 1.5% agarose gel. Amplicons were purified using Expin PCR SV kit (GeneAll) following manufacturer instructions and sequenced using an ABI 3730xl DNA Analyzer (ThermoFisher Scientific) at CPOS.

#### Phylogenetic Analysis

The gene sequences RNA-dependent-RNA-polymerase of the coronaviruses identified in our rodent samples were aligned with selected previously published coronavirus sequences (largely alphacoronavirus and betacoronavirus) using MAFFT v7.273 (*2*). The multiple alignment was manually checked for accuracy and poor gap regions were trimmed. A phylogenetic tree was estimated using the maximum likelihood method and GTRGAMMA substitution model implemented in RAxML v8.2.12 (*3*). One hundred multiple inferences were performed, and the best tree was selected for comparison with 500 bootstrap replicates.

#### Serology

Detection of antibodies with activity against the SARS-CoV-2 spike protein was performed on heat-inactivated serum using the WANTAI SARS-CoV-2 Ab ELISA Diagnostic Kit (Beijing Wantai Biologic Pharmacy Enterprise Co., Ltd, https://www.ystwt.cn), a double-antigen binding assay for detection of total antibodies to SARS-CoV-2, following manufacturer instructions. Absorbance was measured at 450nm with a reference wavelength set at 620 nm using a FilterMax F5 multimode microplate reader. Each sample was tested twice following manufacturer recommendations and established cutoff values for positive (absorbance/cutoff value >1.1) or borderline samples (absorbance/cutoff value 0.9–1.1). Test results were considered valid if the absorbance of the 2 internal positive controls were ≥0.19 and if the absorbance of the 3 internal negative controls were ≤0.1. For each test, cutoff values were calculated as mean absorbance of the 3 internal negative controls (use 0.03 if <0.03) plus 0.16. In the case of unambiguous positive (i.e., both replicates showing absorbance/cutoff values >1.1) and inconclusive (i.e., only 1 of the 2 replicates giving a positive absorbance/cutoff ratio >1.1, or with 1 or both replicates giving a borderline absorbance/cutoff ratio of 0.9–1.1) results from ELISA, the samples were further tested using an in-house SARS-CoV-2 surrogate virus neutralization test (sVNT) as described elsewhere (*4*,*5*). This method has been validated not to cross-react with serum of rodents containing antibodies against murine hepatitis virus as well as serum containing antibodies to several other epizootic alpha- and betacoronaviruses (*4*). An in-

house plaque-reduction neutralization test described elsewhere (*4*) was used to further investigate the sVNT-positive finding.

Fifty rodent serum samples collected in 2008 were examined by ELISA as a pre-COVID-19 biologic control. Their resulting absorbance/cutoff values were between −0.096 to 2.070. Two of the pre-COVID-19 serum samples showed inconclusive results in the ELISA; none exhibited unambiguously positive results.

## References


1. Sabir JSM, Lam TT-Y, Ahmed MMM, Li L, Shen Y, Abo-Aba SE, et al. Co-circulation of three camel coronavirus species and recombination of MERS-CoVs in Saudi Arabia. Science. 2016;351:81–4. PubMed https://doi.org/10.1126/science.aac8608
2. Katoh K, Standley DM. MAFFT multiple sequence alignment software version 7: improvements in performance and usability. Mol Biol Evol. 2013;30:772–80. PubMed https://doi.org/10.1093/molbev/mst010
3. Stamatakis A. RAxML version 8: a tool for phylogenetic analysis and post-analysis of large phylogenies. Bioinformatics. 2014;30:1312–3. PubMed https://doi.org/10.1093/bioinformatics/btu033
4. Perera RAPM, Ko R, Tsang OTY, Hui DSC, Kwan MYM, Brackman CJ, et al. Evaluation of a SARS-CoV-2 surrogate virus neutralization test for detection of antibody in human, canine, cat, and hamster sera. J Clin Microbiol. 2021;59:e02504–20. PubMed https://doi.org/10.1128/JCM.02504-20
5. Tan CW, Chia WN, Qin X, Liu P, Chen MI, Tiu C, et al. A SARS-CoV-2 surrogate virus neutralization test based on antibody-mediated blockage of ACE2-spike protein-protein interaction. Nat Biotechnol. 2020;38:1073–8. PubMed https://doi.org/10.1038/s41587-020-0631-z

**Appendix Table 1.** The number of rodents (*Rattus* spp.) sampled from urban areas within Hong Kong

| District | Locality | No., *R. norvegicus* sampled alive; dead | No., *R. tanezumi* sampled alive; dead |
|---|---|---|---|
| Hong Kong Island | | | |
| Central and Western | Sai Wan | 0, 0 | 1, 0 |
| Kowloon | | | |
| Kowloon City | Hung Hom | 4, 0 | 0, 0 |
| | Kai Tak | 1, 0 | 0, 0 |
| | Kowloon city | 0, 0 | 1, 0 |
| | To Kwa Wan | 46, 6 | 3, 0 |
| Kwun Tong | Kwun Tong | 4, 0 | 1, 0 |
| | Ngau Tau Kok | 4, 0 | 1, 0 |
| Sham Shui Po | Sham Shui Po | 46, 2 | 2, 0 |
| Yau Tsim Mong | Ho Man Tin | 1, 0 | 0, 0 |
| | Jordan | 1, 0 | 0, 0 |
| | Tsim Sha Tsui | 15, 3 | 5 |
| | Yau Ma Tei | *47, 9 | 13, 4 |
| | Unknown | 0, 0 | 1, 0 |
| New territories | | | |
| Kwai Tsing | Kwai Chung | 9, 0 | 0, 0 |
| North | Sheung Shui | 9, 0 | 0, 0 |
| Tuen Mun | Tuen Mun | 2, 0 | 0, 0 |
| Total | | 189, 20 | 28, 4 |

*Single seropositive rat, Rat-213, captured in this location.

**Appendix Table 2.** PCR detection of SARS-CoV-2 and other coronaviruses in rodents from Hong Kong*

| Sample types | SARS-CoV-2 positive | UniCoV positive |
|---|---|---|
| Body surface swab | 0/217 | 2/217 |
| Oropharyngeal swab | 0/217 | 3/217 |
| Rectal swab | 0/217 | 3/217 |
| Blood | 0/194 | 1/194 |
| Lymph node | 0/186 | 1/186 |
| Lung | 0/186 | 2/186 |
| Trachea | 0/186 | 2/186 |
| Small intestine | 0/186 | 1/186 |
| Urine | 0/94 | 0/94 |

*SARS-CoV-2, severe acute respiratory syndrome coronavirus 2; UniCoV, universal coronavirus.

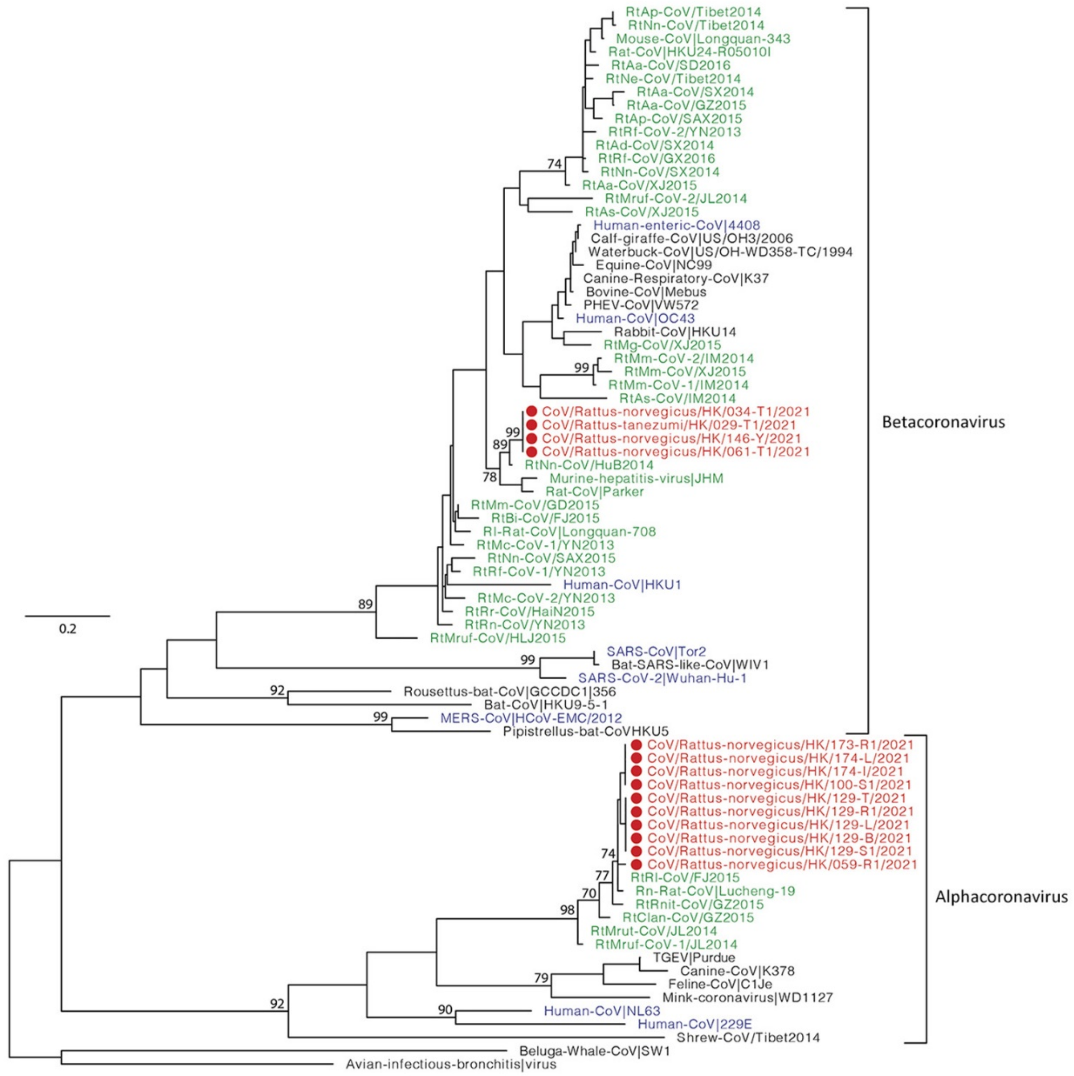


**Appendix Figure 1.** Phylogenetic tree showing the evolutionary relationship of alphacoronavirus and betacoronavirus found in the rodent samples collected February–May 2021. The tree was estimated based on the universal coronavirus primers amplicon region (located within the RNA-dependent-RNA-polymerase) using a maximum likelihood method. Rodent samples reported in this study are indicated with dots and in red (i.e., 14 specimens from 9 individual rodents; details in Appendix Table 2; Rat-034 tracheal sample was omitted from the tree due to poor sequencing quality). Reference coronavirus sequences from humans are indicated in blue and from rodents in green. Bootstrap support values (percentage; from 500 bootstrap replicates) for selected lineages are shown.

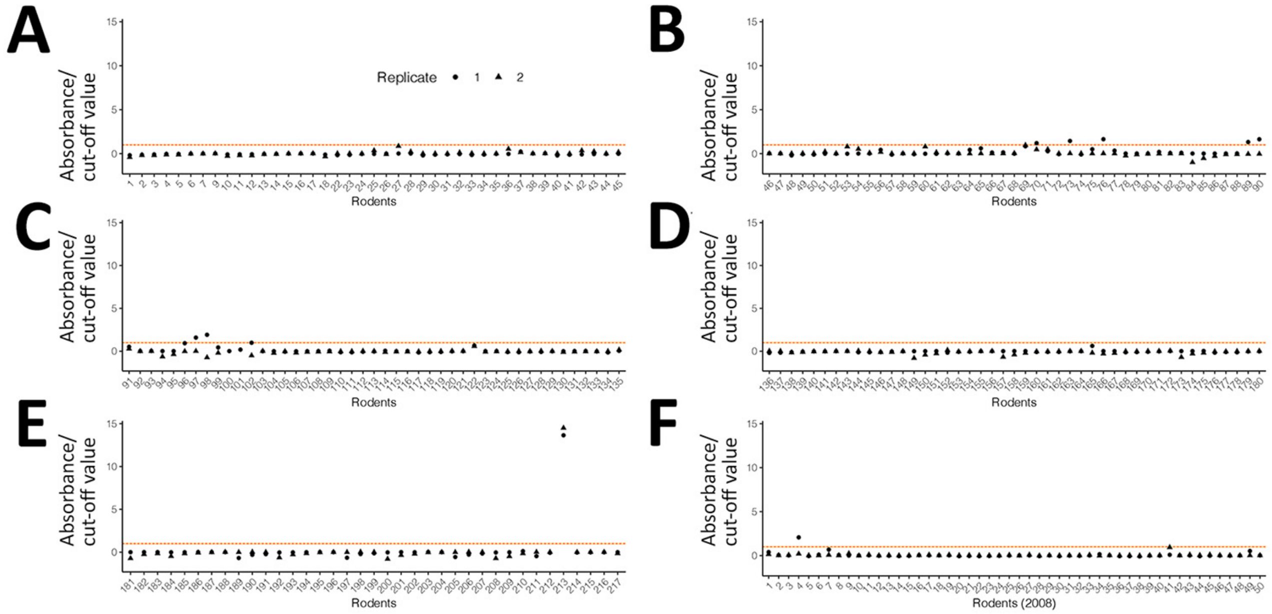


**Appendix Figure 2.** ELISA of the 213 rodent serum samples collected in 2021 (A, B, C, D, and E) and the 50 rodent serum samples collected in 2008 that we used as pre-COVID-19 biologic controls (F). Absorbance/cutoff value is interpreted as negative if <0.9, borderline if 0.9–1.1, and seropositive if >1.1. Each serum sample was tested twice, and the rodent considered unambiguously positive if both replicates were seropositive. The red dashed line represents the seropositivity threshold (absorbance/cutoff: >1.1) and the orange-shaded area represents borderline samples (absorbance/cutoff: 0.9–1.1).
